# Myocardial infarction in a 33-year-old with inflammatory bowel disease: a case report

**DOI:** 10.1186/s12872-023-03284-x

**Published:** 2023-05-16

**Authors:** Christopher Paul Bengel, Denisa Müller-Gastell, Bassam Al-Najjar, Irina Cherednichenko, Rifat Kacapor

**Affiliations:** Department of Cardiology, Frankfurt-Main-Taunus Clinics, Bad Soden Hospital, Kronberger Str. 36, 65812 Bad Soden, Germany

**Keywords:** Myocardial Infarction, MINOCA, STEMI, Inflammation, Inflammatory Bowel Disease, Case report

## Abstract

**Background:**

ST elevation myocardial infarction is defined as acute myocardial injury with necrosis due to myocardial ischemia. The frequent cause is thrombotic occlusion of atherosclerotic coronary arteries. In particular situations, thromboembolism can cause myocardial infarction in patients with normal coronary arteries.

**Case presentation:**

We report a particular case of myocardial infarction in a young, previously healthy patient with non-atherosclerotic coronary arteries and inflammatory bowel disease. Although we performed an extensive work up, no clear pathophysiological cause could be diagnosed. Most likely, myocardial infarction was associated with a hypercoagulative state related to systemic inflammation.

**Conclusion:**

The mechanisms of coagulation disturbances in the context of acute and chronic inflammation are not yet fully understood. A better understanding of cardiovascular events in patients with inflammatory bowel disease might lead to new treatment approaches of cardiovascular disease.

**Supplementary Information:**

The online version contains supplementary material available at 10.1186/s12872-023-03284-x.

## Background

In the young population, acute myocardial infarction is rare. A higher risk of cardiovascular events is associated with common risk factors [1]. Presentation as MINOCA is also more common, as is the presence of single vessel disease [2]. The most common cause is thrombotic occlusion of atherosclerotic coronary arteries after plaque rupture. In certain cases, coronary angiograms do not reveal angiographically significant atherosclerosis, and despite extensive investigations, the underlying cause remains unclear. A link is thought to exist between inflammatory diseases and acute coronary syndromes [3–5]. Only few reports have been discussing cardiovascular events in young patients with inflammatory bowel disease (IBD) [6–8], and comprehensive pathophysiological explanations are usually not available.

We report the rare case of a previously healthy young woman with acute ST-elevation myocardial infarction in an acute flare of a previously unknown ulcerative colitis.

## Case presentation

**Fig. 1:**
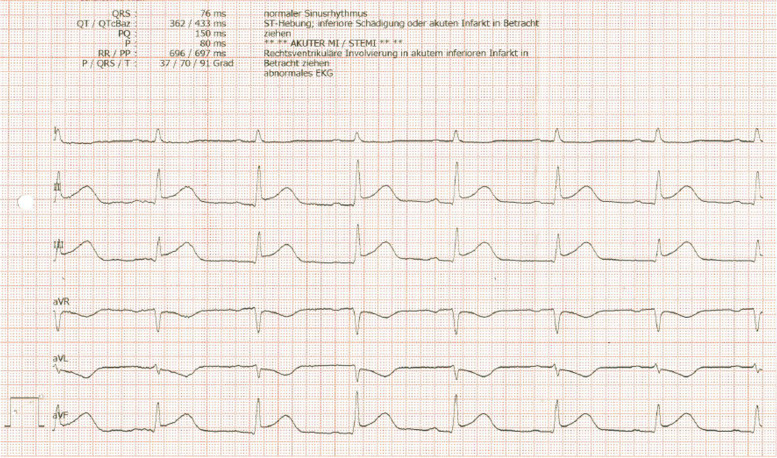
Electrocardiogram, standard limb leads, showing ST segment elevation in leads II, III, aVF

**Fig. 2:**
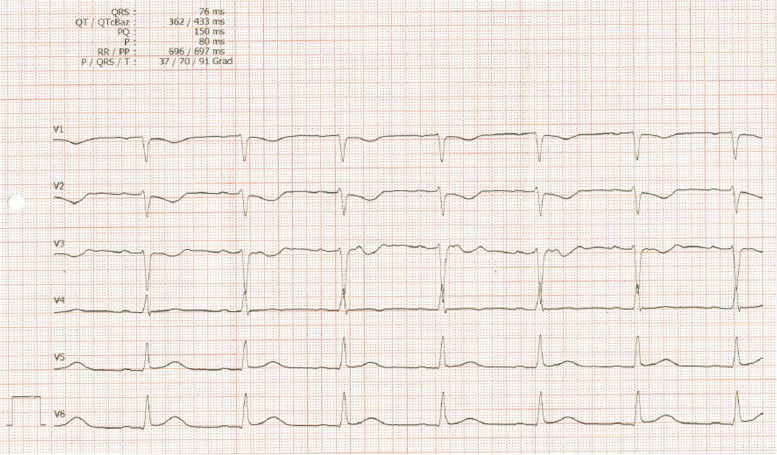
Electrocardiogram, chest leads, showing inverted T-waves in leads V1-V3

**Fig. 3:**
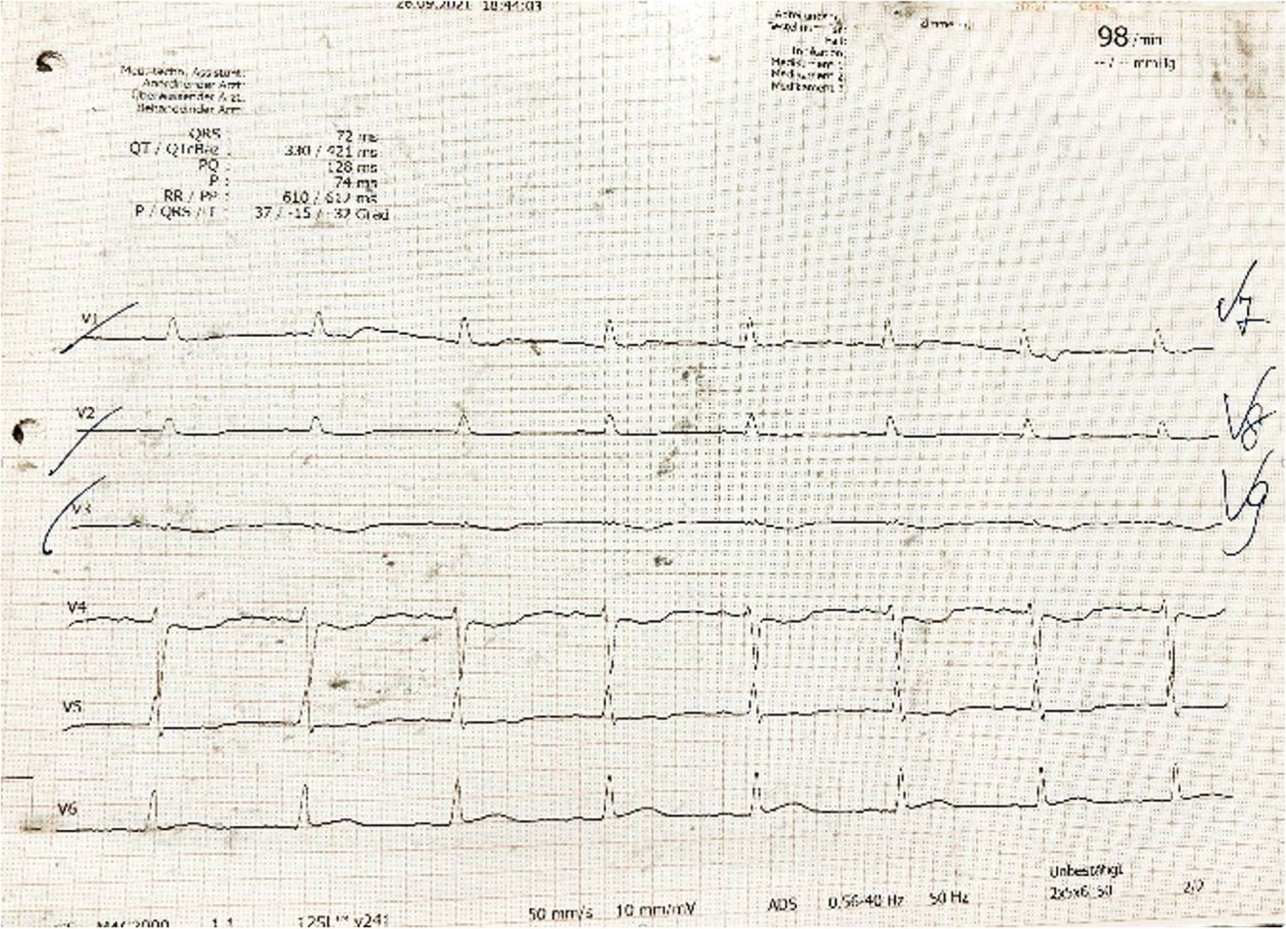
Electrocardiogram, chest leads, showing flattening of T-waves in leads V7-V8 and inverted T-waves in V9

A 33-year-old previously healthy woman presented with bloody diarrhea and nausea. An ECG was performed as part of the routine procedures of the emergency room. The electrocardiogram revealed a myocardial infarction with ST segment elevation in leads II, III, aVF and inverted T-waves in leads V1-V3 (Figs. 1, 2) as well as a flattening of T-waves in V7-V8 and inverted T-waves in V9 (Fig. 3). The patient did not state to have any chest pain.

She was not taking any medication. On initial evaluation, her blood pressure was 94/76 mmHg, heart rate was 98 bpm. Upon cardiac auscultation, there were no audible murmurs, and her lungs were clear. No oedema and no signs of congestion were noted. There was slight tenderness on palpation throughout the abdomen.

Laboratory data showed a significant increase of high-sensitivity cardiac troponin-T (578 pg/mL; normal: < 14 pg/mL), creatine kinase (317 U/L; normal: < 167 U/L), creatine kinase-MB (52 U/L; normal: < 24 U/L), C-reactive protein (191 mg/L; normal: 0–5 mg/L), lactate dehydrogenase (236 U/L; normal: 135–214 U/L) and D-dimer (16.95 µg/mL; normal: < 0.5 µg/mL). Other abnormal laboratory data included a decreased platelet count of (85 G/L; normal: 150 – 370 G/L).

Hemoglobin (14 g/dL; normal: 12 – 15.6 g/dL) as well as coagulation factors showed no abnormality. Further laboratory results, including lipids, glucose and electrolytes were also within the normal range.

An urgent coronary angiography revealed a thrombotic occlusion of the periphery of the left circumflex coronary artery (Video 1). Pre-treatment with acetylsalicylic acid was not given, however the patient received 5000 IU heparin. Due to the localization of the thrombus in the vascular periphery, stent implantation was not feasible. Probing with a wire and a non-inflated percutaneous coronary intervention balloon was performed, but distal perfusion was not restored. The patient received a bolus of Tirofiban, however no permanent infusion of the medication was given due to the persisting intestinal bleeding. Dual antiplatelet therapy with acetylsalycylic acid 100 mg and ticagrelor 90 mg b.i.d. was initiated according to the ESC guidelines [9].

The echocardiographic examination revealed a normal left ventricular ejection fraction (Video 2). No segmental ventricular wall motion abnormalities and no ventricular thrombus were observed. In the transesophageal echocardiography (TEE) no intracardiac thrombus and no structural anomaly could be detected. Due to low imaging quality of the first study, we repeated the TEE study. Neither a thrombus nor a patent foramen ovale, also using a bubble test, could be detected in the additional transesophageal echocardiography.

**Fig. 4:**
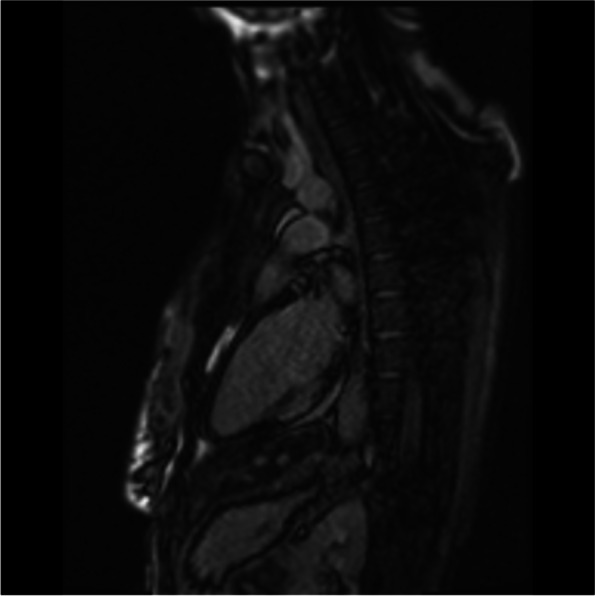
Cardiac MRI, inverse recovery LGE sequence, long axis view, showing transmural late gadolinium enhancement of the mid inferior segment in an ischemic distribution pattern

**Fig. 5:**
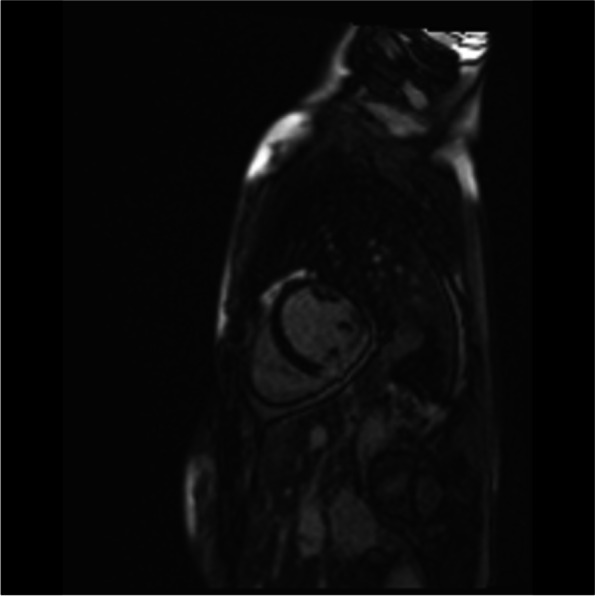
Cardiac MRI inverse recovery LGE sequence, midventricular short-axis view, showing transmural late gadolinium enhancement of the mid inferior segment in an ischemic distribution pattern

**Fig. 6:**
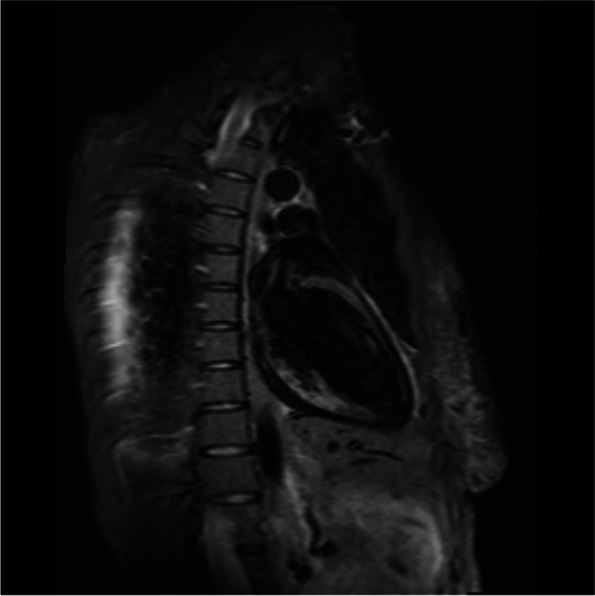
Cardiac MRI, T2-weighted stir sequence, long-axis view, showing an increased signal intensity of the mid inferior and basal inferior segment

**Fig. 7:**
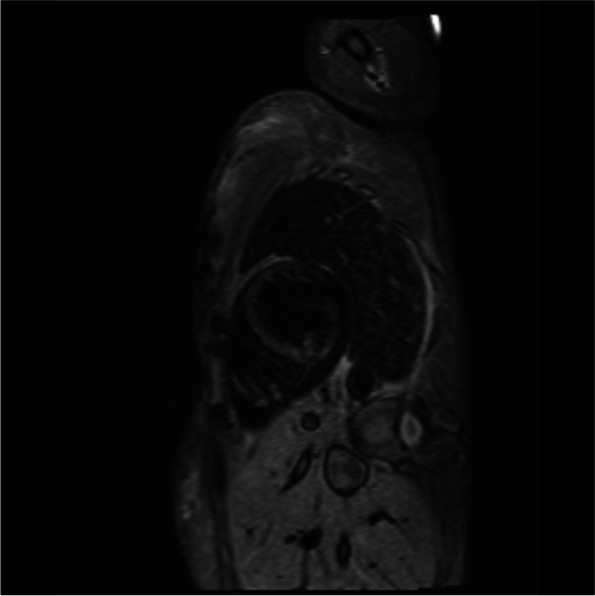
Cardiac MRI, T2-weighted stir sequence, midventricular short-axis view, showing an increased signal intensity of the mid inferior segment

A cardiac magnetic resonance imaging (MRI) was carried out. There was transmural late gadolinium enhancement (LGE) of the basal and mid inferior left ventricular segments, corresponding to an ischaemic distribution pattern, as well as increased signal intensity of the basal and mid inferior left ventricular segments (Figs. 4, 5, 6 and 7). Cine sequences revealed a moderately reduced LV systolic function with hypokinesis of the inferior myocardial segments and an LVEF of 46% (Video 3).There was no evidence of a left ventricular thrombus. While no abnormalities were detected in echocardiography, cardiac MRI showed regional wall motion abnormalities, probably due to better definition of the endocardium as well as limited echocardiographic assessability due to tachycardia during echocardiography.

**Fig. 8:**
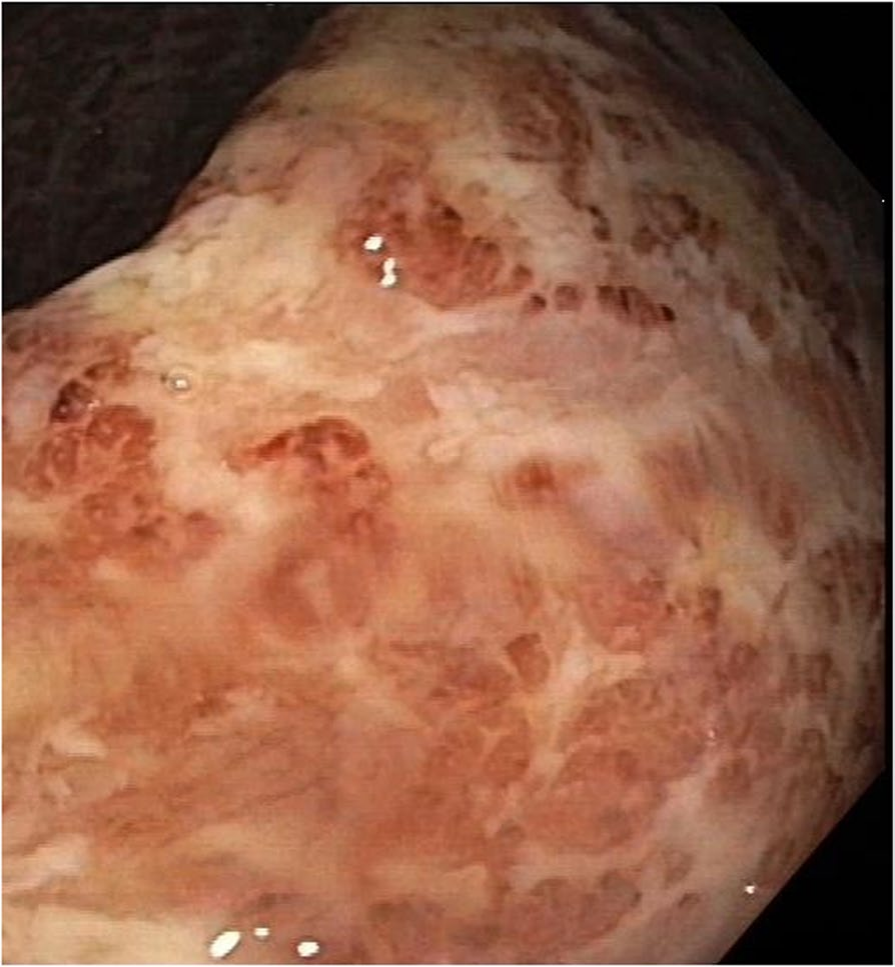
Colonoscopy showing mucosal ulcerations extending into deeper layers and loss of vascular pattern

Further diagnostic work up identified a highly active ulcerative pancolitis (Fig. 8). An infectious cause of the dysentery was ruled out. Hepatitis, human immunodeficiency virus, cytomegaly virus and tuberculosis were ruled out.

An extensive diagnostic work up for cardiac thromboembolism was performed [10].

Although we found evidence of vascular thrombosis on the coronary angiogram, there was no history of pregnancy morbidity and the patient tested negative for antiphospholipid antibodies, thus the revised Sapporo Criteria for Antiphospholipid Syndrome (APS) were not met, rendering a primary antiphospholipid syndrome as cause of the myocardial infarction very improbable [11]. Clinical criteria for Systemic Lupus Erythematosus (SLE) were absent, and there was a negative test result for Antinuclear Antibodies (ANA), also eliminating an SLE-associated antiphospholipid syndrome as an explanation [11].

A test for factor V Leiden mutation or a Factor II-20210A mutation was negative, thus these types of hereditary hypercoagulability were not taken into consideration as a differential cause for the myocardial infarction [10].

In the absence of clinical clues or coronary malformations, mid-size vessel vasculitis as a cause of the myocardial infarction was highly unlikely. In addition, there were negative results for Anti-neutrophil cytoplasmic antibodies (ANCAs), making an ANCA-associated vasculitis as a cause of the myocardial infarction even more improbable [12].

A coagulopathy was also conceivable in the context of a COVID-19 infection [13]. We ruled this possibility out through a PCR Test.

Atrial fibrillation as cause [10] of cardiac embolism was not recorded in a 7-day clinical monitoring period.

Potentially thrombogenic medication was not present. The patient was not taking oral contraceptives or anti-inflammatory drugs [14].

Thromboembolism in the context of acute bleeding was suggestive of disseminated intravascular coagulation (DIC). The ISTH Criteria for Disseminated Intravascular Coagulation [15] were not suggestive of overt DIC but a non-overt DIC could not be ruled out.

Inflammatory bowel disease (IBD) was first diagnosed in our patient and she had been without treatment.

Inflammatory markers were strongly elevated (C-reactive protein 191 mg/L; [normal: 0–5 mg/L]). We considered this to be an expression of an acute flare of the ulcerative colitis. The patient was treated with prednisolone 60 mg once daily and mesalazine 3 g once daily. Because of the lack of macroscopic improvement, we also initiated immune-modulating therapy with infliximab 300 mg I.V. Although the use of corticosteroids may be a risk factor for mechanical complications of myocardial infarction [16], we considered their use mandatory because of the high inflammatory IBD activity.

The developement was complicated by difficult-to-control lower gastrointestinal bleeding with a decreased hemoglobin value of 5.6 g/dL in the setting of ulcerative colitis and dual antiplatelet therapy with acetylsalycylic acid 100 mg and ticagrelor 90 mg b.i.d. The patient received two blood transfusions and the antithrombotic therapy was initially switched to to acetylsalycylic acid 100 mg and enoxaparin 60 mg once daily, but only after administration of infliximab and further modification of anticoagulative and anti-aggregatory therapy to apixaban 2,5 mg b.i.d. the lower gastrointestinal bleeding stopped.

On the day of discharge, the patient stated to now experience chest pain. A pericardial effusion was seen on the subsequent echocardiogram. We interpreted this in the context of Dressler syndrome and administered a therapy with colchicine for three months. Furthermore, we suggested to the patient that she should visit a specialized coagulation outpatient clinic. We scheduled a follow up with an ambulant care physician where she received the second dose of infliximab 300 mg I.V. two weeks later.

## Discussion and conclusions

We report the case of a 33-year-old previously healthy woman with acute ST-elevation myocardial infarction. Coronary angiography revealed a thrombotic occlusion of the left posterolateral and 2^nd^ obtuse marginal branch of the circumflex coronary artery. At the same time, there was a lower gastrointestinal hemorrhage. Both occurred in an acute flare of a previously unknown ulcerative colitis.

The occurrence of bleeding and coronary thrombosis at the same time poses a therapeutic dilemma: Firstly, one would pursue an aggressive management to prevent further thrombotic events. This approach was hampered by repeated bleeding events. Secondly, there was therapeutic insecurity on the best approach regarding the anticoagulatory therapy. Antiplatelet therapy in the context of STEMI was indicated according to international guidelines and recommendations [9, 10]. Inhibition of the coagulation cascade would be the preferred treatment for cardiac embolism [17]. A multifactorial anticoagulation therapy (e.g. vitamin K antagonists) would likely be able to prevent further thrombotic events in the most effective way, as has been shown in Antiphospholipid Syndrome [18]. However, this option was not considered given the concern of exacerbation of the bleeding. Ultimately, an adjustment of antithrombotic therapy to reduced-dose directly acting oral anticoagulants in conjunction with anti-inflammatory treatment of the ulcerative colitis resulted in remission.

Acute myocardial infarctions in young people are rare and different [2]. A high risk of cardiovascular events is associated with diabetes, hypertension, hyperlipidemia and smoking for the overall population, while cigarette smoking, hyperlipidemia and family history of coronary artery disease are prominent risk factors for acute myocardial infarctions in younger patients [1]. Our young patient however did neither exhibit any of the aforementioned risk factors nor atherosclerosis in the coronary angiogram. The criteria of a myocardial infarction with non-obstructive coronary arteries (MINOCA) [10] were applied in the work up. Although we performed an extensive work up, no evidence was found for the common causes of a myocardial infarction of embolic etiology such as paradoxical or cardiac embolism, coagulopathy, vasculitis, antiphospholipid syndrome, or rhythm disorders [10].

Due to the limited number of reports, a comprehensive comparison with similar cases is difficult. Yet on comparing our patient’s case with previously reported cases of myocardial infarction in the context of IBD, we noted similarities in terms of patient characteristics, clinical course and IBD disease activity. There was a predominance of female patients. Almost all suffered from an acute flare of IBD. Coronary arteries were non-obstructive and non-stenotic in the majority of cases, and there was evidence of myocardial infarction due to non-atherosclerotic coronary thrombosis [6–8]. Acute myocardial infarction and acute lower gastrointestinal bleeding were present at the same time [7, 8].

A link is thought to exist between inflammatory diseases, including inflammatory bowel disease (IBD) and cardiovascular disease [3–5]. Several pathophysiological mechanisms could be proposed as explanations, but all remain speculative:

Medication with corticosteroids was found to be aggravating the cardiovascular conditions of IBD patients [5]. Conversely, treatment with corticosteroids can be an indicator of high inflammatory activity of the underlying IBD instead of actually increasing cardiovascular risk. Since we had diagnosed the patient with ulcerative colitis for the first time, she had not been treated with corticosteroids until then.

Thromboembolic risk may be exaggerated by the use of oral contraception. This might explain the increased risk for acute thromboembolic complications in young women with IBD. Yet our patient was not taking oral contraceptives. Thus, adverse effects of these medications could be ruled out as a pathophysiological explanation.

DIC of the “bleeding type” is conceivable [15], although, as mentioned above, it could not be proven.

Another possible mechanism might be endothelial dysfunction: A thrombogenic state of the coronary endothelium might be triggered by intensely elevated inflammatory cytokines in an acute flare of IBD. Pathological platelet activity triggered by the acute inflammatory reaction of the endothelium may be a possible cause of spontaneous coronary thrombosis [4, 5].

Although we found no anomaly predisposing to paradoxical embolism or cardiac embolism, it is conceivable that an intracardiac thrombus embolized into the coronary arteries, especially in light of the distal obstruction of several coronary branches as shown in the patient’s angiogram [10], (Video 1).

Even if this cannot be proven by the available data, the clinical presentation, the evidence of non-stenotic coronary arteries with evidence of intracoronary thrombus and the positive DIC score speak for a thromboembolic event, although a definitive diagnosis could not be made.

Among the uncertainties that accompany this case report, the following limitations should be highlighted: Intravascular ultrasound (IVUS) would have been very helpful to further clarify the pathophysiology. However, it was not applied because of concern for vascular injury with very small vessel diameters. Furthermore, since provocative testing for coronary vasospasm was not performed, we cannot make any reliable statement about a possible vasospasm. However, vasospasm is also not a likely mechanism in embolic infarction. Although atrial fibrillation was not present in the monitoring period, this does not exclude paroxysmal atrial fibrillation as an etiology of cardiac embolism.

Overall, we assume that the myocardial infarction was caused by a disturbance in the balance of the coagulation system, which resulted from the acute episode of IBD. We assumed a hypercoagulable state due to acute systemic inflammation.

More research is needed to clarify the mechanism of coagulation disturbances in the context of acute and chronic inflammation, as the number of patients with IBD is ever increasing [4, 5]. Inflammation may have deleterious effects on the coagulation system and the risk of thromboembolism and bleeding can be greatly increased by inflammatory conditions.

As obvious as it may seem, all patients in the emergency room must undergo an ECG. Without it, our patient's heart attack could easily have been missed.

## Supplementary Information

**Additional file 1: Video 1.** Coronary angiography, RAO caudal view, showing non-stenotic coronary arteries and a thrombotic occlusion of the periphery of the left posterolateral and 2^nd^ obtuse marginal branch of the circumflex coronary artery.

**Additional file 2: Video 2. **Transthoracic echocardiogram, apical 4-chamber view, showing normal left ventricular ejection fraction.

**Additional file 3: Video 3.** Cardiac MRT cine, long axis 2 chamber view.

## Data Availability

All data generated or analysed during this study are included in this published article.

## Abbreviations

ANA: Antinuclear Antibodies
ANCA: Anti-neutrophil cytoplasmic antibodies
APS: Antiphospholipid Syndrome
DIC: Disseminated intravascular coagulation (DIC).
ECG: Electrocardiogram
ESC: European Society of Cardiology
IBD: Inflammatory bowel disease
IVUS: Intravascular ultrasound
LGE: Late gadolinium enhancement
MINOCA: Myocardial infarction with non-obstructive coronary arteries
MRI: Magnetic resonance imaging
SLE: Systemic lupus erythematosus
STEMI: ST elevation myocardial infarction
TEE: Transesophageal echocardiography
